# Social behavior in 16p11.2 and 22q11.2 copy number variations: Insights from mice and humans

**DOI:** 10.1111/gbb.12787

**Published:** 2021-12-09

**Authors:** Arianna Benedetti, Cinzia Molent, Weronika Barcik, Francesco Papaleo

**Affiliations:** ^1^ Genetics of Cognition laboratory, Neuroscience area Istituto Italiano di Tecnologia Genoa Italy; ^2^ CNRS, GREDEG Université Côte d'Azur Nice France; ^3^ Dipartimento di Medicina Sperimentale (Di. Mes) Università degli Studi di Genova Genoa Italy; ^4^ Department of Neurosciences and Mental Health Fondazione IRCCS Ca' Granda Ospedale Maggiore Policlinico Milan Italy

**Keywords:** 16p11.2, 22q11.2, autism spectrum disorder, development, schizophrenia

## Abstract

Genetic 16p11.2 and 22q11.2 deletions and duplications in humans may alter behavioral developmental trajectories increasing the risk of autism and schizophrenia spectrum disorders, and of attention‐deficit/hyperactivity disorder. In this review, we will concentrate on 16p11.2 and 22q11.2 deletions' effects on social functioning, beyond diagnostic categorization. We highlight diagnostic and social sub‐constructs discrepancies. Notably, we contrast evidence from human studies with social profiling performed in several mouse models mimicking 16p11.2 and 22q11.2 deletion syndromes. Given the complexity of social behavior, there is a need to assess distinct social processes. This will be important to better understand the biology underlying such genetic‐dependent dysfunctions, as well as to give perspective on how therapeutic strategies can be improved. Bridges and divergent points between human and mouse studies are highlighted. Overall, we give challenges and future perspectives to sort the genetics of social heterogeneity.

**Abbreviations**
16p‐DEL: carriers of the proximal 16p11.2 deletion
22q‐DEL: carriers of the 22q11.2 deletion
ADHD: attention‐deficit/hyperactivity disorder
ADI: autism diagnostic interview
ADOS: autism diagnostic observation schedule
ASD: autism spectrum disorder
BP: breakpoint
CBCL: child behavior checklist
CNVs: copy number variations
DR: dorsal raphe
GF: germ free
HT: hydroxytryptemine
iASD: idiopathic autism spectrum disorder
NDD: neurodevelopmental disorders
PFC: prefrontal cortex
PND: postnatal day
SCQ: social communication questionnaire
SRS: social responsiveness scale
TASIT: the awareness of social inference test
USV: ultra sound vocalization
VABS: vineland adaptive behavior scale
WT: wild type

## 1 INTRODUCTION

Copy number variations (CNVs) are submicroscopic deletions or duplications of DNA segments. CNVs have consistently been implicated as vulnerability factors for neurodevelopmental disorders.^1^ CNVs genetic alterations can range from 10^2^ to 10^6^ base pairs and consist of complex alterations of homologous sequences at multiple sites in the genome or of simple gains or losses of genetic information.^2^ Consequently, they can impact multiple protein‐coding genes and regulatory regions.^3^

The total prevalence of recurrent neurodevelopmental disorders associated with CNVs (duplications and deletions) has been estimated in live‐born children to 0.48% that is, ~1 in 200 newborns has either a deletion or duplication.^4^, ^5^ Noteworthy, there are hotspots for CNVs in the human genome.^6^ In this review, we will focus on 16p11.2 and 22q11.2 genetic loci that are among the most common and most studied CNVs with high penetrance to neuropsychiatric disorders.^5^, ^7^ CNVs on 16p11.2 and 22q11.2 genetic loci are associated with increased risk of developing schizophrenia, attention‐deficit/hyperactivity disorder (ADHD) and autism spectrum disorder (ASD), compared with the general population. More specifically, CNVs at the proximal region of the 16p11.2 locus refer to the deletion or duplication of a segment of 600 kb with breakpoints (BP) at 29.5 and 30.1 Mb (BP4‐BP5).^8^ 22q11.2 microdeletions (also named as Di George syndrome and velocardiofacial syndrome), refer to the deletions of a segment of 1.5–3 Mb on the long arm of chromosome 22.^9^, ^10^, ^11^ Duplication cases are less frequently reported and still less described, with reported phenotypes that can range from mild cognitive impairment to apparent normal cognition.^12^, ^13^

**FIGURE 1:**
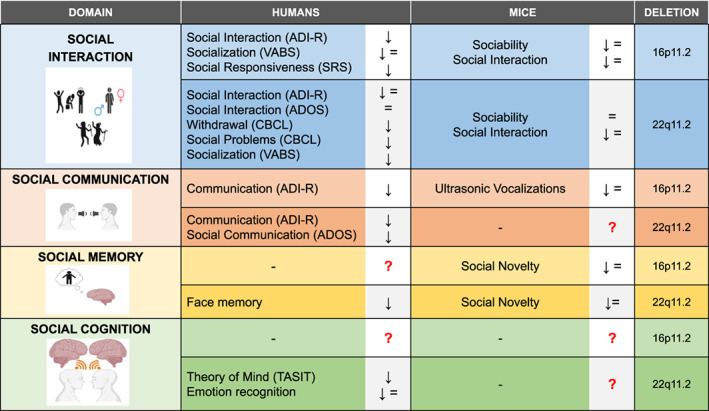
Summary of the main findings in humans and in mice models of 16p11.2 and 22q11.2 deletions. For humans, arrows indicate presence (↓) or absence (=) of problems, identified through the mentioned instruments. The arrows are therefore not directly referring to higher or lower raw scoring of the instrument. For mice, symbols represent the trend of mice performances during the tasks used for assessing the social behavior, in comparison with their controls. Specifically, we used (↓) or (=) to indicate, respectively, the decrease or the comparability of tests results deriving from 16p11.2 and 22q11.2 deletion mice with those shown by control mice. Notably, the variability of results within each social item could be a consequence of genetic and/or environmental factors. Refer to the text for more details. (Pictures created with BioRender.com)

Here, we will concentrate on social behavioral alterations associated with the 16p11.2 and 22q11.2 hemi‐deletion syndromes, and we will contrast the inherent available data collected in humans and in mouse models. Our goals are to (i) better integrate human observational studies with the biological findings derived from animal research; (ii) highlight similarities and distinctions between different CNVs conditions. See Figure 1 for a summary. We believe this might ultimately serve as a powerful platform to advance our knowledge of social processes and suggest more efficient strategies to ameliorate social dysfunctions.

A recent review summarized the genotype–phenotype correlations in proximal 16p11.2 deletions and duplications in humans and mice.^14^ A comprehensive description of the behavioral features of mouse models and of the human phenotype from a diagnostic perspective were reported, but the social component was only briefly described. Similarly, other reviews have compared ASD‐ and schizophrenia‐relevant phenotypes in humans and mouse models of 22q11.2 CNVs,^15^, ^16^, ^17^ or the impact of specific single genes contributing to these developmental disorders.^18^ Moreover, Jonas et al. have provided an overview of neuropsychiatric implications of 22q11.2 deletion describing human and mouse studies.^19^ However, in all cases, the social phenotypes have only been marginally characterized and addressed. We aim to provide a critical review of the literature on 16p11.2 and 22q11.2 CNVs and their influence on distinct aspects of social processes that might inform on future studies beyond more strict psychiatric classification.

## 2 METHODS

**FIGURE 2:**
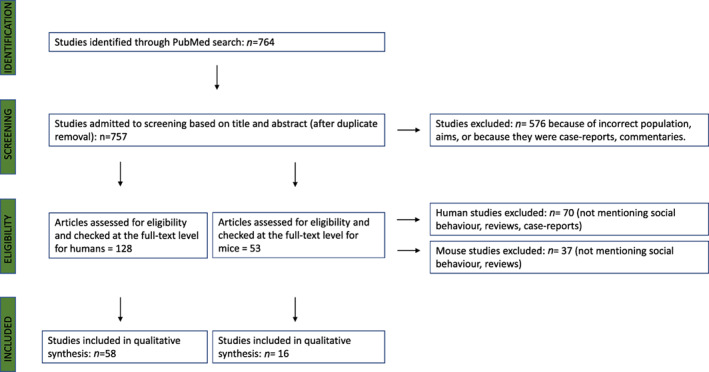
Flow chart summarizing the selection process of papers included in the review. The full process of identification, screening, eligibility and inclusion of the literature papers relevant for the purposes of this review is summarized in the flow chart. For each step, the number of papers that were included or excluded is reported, for both humans and mouse studies. The main reasons for exclusion are mentioned for each step

We collected the most relevant literature papers from PubMed by using the following search string (“16p11.2” OR “22q11.2”) AND “deletion” AND (“social” OR “sociability” OR “emotion” OR “emotional” OR “face” OR “recognition” OR “cognition” OR “cognitive” OR “behavior” OR “behavior”). For description of the selection process, please see Figure 2. We included all search results covering human and mouse studies. Case‐reports, commentaries and reviews, were excluded. Only studies including information about 16p11.2 and 22q11.2 deletions and social or behavioral measures were included.

### 2.1 Preliminary considerations for studies in humans

Even though 16p11.2 and 22q11.2 CNVs are associated with a high risk of developing specific neurodevelopmental disorders and other psychiatric conditions, not all carriers with these CNVs have impairments that reach the threshold for a psychiatric diagnosis. In fact, carriers of CNVs can present sub‐threshold abnormalities in specific social behavioral sub‐domains, which do not require clinical attention. In depth characterization of abnormalities in these sub‐constructs could improve our understanding of their psychopathological trajectories, which may lead to a better understanding of the underlying biological mechanisms, and consequently, to an improved design of animal studies.

Attempting to describe how each social sub‐construct could be impaired in CNVs carriers comes with methodological issues. The biggest problem, which we cannot overcome in the context of this review, is that studies on CNVs have a high risk of an ascertainment bias. The genetic testing is usually performed only on patients with a clinical indication, usually developmental delay. This means that CNVs carriers without strong clinical impairments could be underrepresented, or represented in a different manner in each cohort, depending on the criteria for the access to genetic testing. The only way to solve this issue would be screening for CNVs all newborn children prior to the identification of impairments.

Secondly, CNVs carriers have rarely been studied with the specific aim of characterizing their social profile: in the majority of cases, the assessment of social and behavioral impairments in CNVs is made with the aim of identifying disorders with a categorical diagnostic approach. Thus, most of the available information on social impairments is derived from studies using assessment tools designed for screening and diagnostic purposes. Some of the exceptions are the cognitive batteries often used in the context of magnetic resonance imaging studies, or scales such as The Awareness of Social Inference Test (TASIT), designed to study theory of mind.^20^ Consequently, the most commonly used assessments tools usually individuate thresholds in macro‐areas of impairments in order to support or disprove the diagnosis, rather than describe a social profile of the tested subjects.

The most frequently used tools for characterizing CNVs that carry information on social impairments are the Autism Diagnostic Observation Schedule (ADOS),^21^ the Autism Diagnostic Interview–Revised (ADI‐R),^22^ the Social Responsiveness Scale (SRS),^23^ and the Social Communication Questionnaire (SCQ).^24^ It is important to note that the majority of these are employed in the context of screening and assessment of ASD: this is because diagnosing ASD (but not schizophrenia, or ADHD) requires assessment of social impairments. Typical social impairments observed in ASD involve three social constructs: (1) Socio‐emotional reciprocity (sharing of emotions or interests, initiating or responding to social interactions, such as conversations and social approach); (2) Nonverbal communication used for social interaction (e.g., expressing or understanding social cues such as facial expressivity, eye contact, body language and integrating verbal and nonverbal communication); (3) Ability to develop, maintain and understand relationships (adjusting behavior in a context‐appropriate manner, showing interest in peers, sharing imaginative play with friends).^25^ Consequently, sub‐scales of the diagnostic tools often contain evaluations of the abovementioned social constructs. Another source of indirect information are the scales used for the dimensional assessment of emotional and behavioral problems or for the evaluation of the patient's overall ability to function in neuropsychiatric disorders. Some examples are the Child Behavior Checklist (CBCL)^26^ and Vineland Adaptive Behavior Scale (VABS).^27^ See Table 1 for a summary of the properties of these tools. Overall, even if the purpose of the abovementioned scales is not the assessment of social domain impairments of CNVs carriers, their sub‐scales can provide useful information on specific social constructs which may be impaired. It is important to keep in mind that diagnostic criteria of neuropsychiatric disorders have evolved over time, and that instruments for the assessment have changed as well. For example, while in DSM‐IV and ICD‐10 the “Social Interaction” and the “Communication” constructs were considered separate contributors to the diagnosis of ASD, these two dimensions were collapsed in the DSM‐5^28^ and in the ADOS as well.^21^ This means that the reporting of some sub‐constructs might be obscured in the most recent studies. To overcome these limitations, we grouped results from studies using similar diagnostic assessment tools, to improve homogeneity when describing domains of social impairments.

**TABLE 1:** Summary of scales, and their sub‐scales, with information on social impairments in ADS

Instrument	Sub‐scales	Examples of social features evaluated
**Autism Diagnostic Observation Schedule (ADOS)**	Social‐affective Repetitive behaviors	Social overtures Social responses Initiating and maintaining behavior Eye‐contact Facial expression Gestures Quality of social response Speech and conversation abnormalities Amount of reciprocal communication Imagination
**Autism Diagnostic Interview‐Revised (ADI‐R)**	Social interaction Communication Repetitive behaviors	Social interaction Peer relationships Shared enjoyment Socio‐emotional reciprocity Gesture Imitation and imagination
**Social Responsiveness Scale (SRS)**	Social awareness Social cognition Social communication Social motivation Mannerisms	Awareness: perceiving social cues Cognition: interpreting social cues Communication: expression of social communication Motivation: motivation to initiate and maintain a social contact
**Social Communication Questionnaire (SCQ)**	Reciprocal interaction Communication Restricted interests	Speech and conversation abnormalities Body language expression Facial expression of emotion Interest in other children Social play
**Child Behavior Checklist (CBCL)**	Total problems Externalizing Internalizing Aggressive behavior Anxiety/depression Attention problems Rule breaking problems Social problems Somatic complaints Thought problems Withdrawal	Withdrawal: Shyness, Preference for loneliness, refusal to talk, secretiveness Social Problems: sociability with peers, quality of social interaction (being not liked by peers or teased, preferring company of younger peers), being dependent, being lonely, speech problems, clumsiness
**Vineland Adaptive Behavior Scale (VABS)**	Communication Socialization Daily living skills Motor skills	Communication: Receptive, written, expressive communication Socialization: interpersonal relationships, play and leisure, ability to cope with social rules

### 2.2 Social impairments in human 16p11.2 proximal deletion carriers

Carriers of the proximal 16p11.2 deletion (16p‐DEL) often have significant impairments in the social domain.^29^ In children with this deletion, social impairments can include deficits in imitation, showing interest, playing, and expressing social cues when interacting with peers.^30^ This CNV has been strongly linked with ASD and around 0.50% of the probands with ASD receiving a genetic test carry the 16p11.2 proximal microdeletion.^31^ On the other hand, among 16p‐DEL the prevalence of ASD has been reported to vary from 11%^8^ to 27%^29^, ^30^ when assessed with both the ADOS and the ADI‐R, and to vary from 22%^32^ to 43%^33^ when assessed with the ADI‐R alone.

Within the 16p‐DEL population, specific construct alterations have been described. Excluding the 16p‐DELs diagnosed with ASD, 60% of the remaining 16p‐DEL presented impairments in the ADI‐R Social Interaction sub‐domain, and 73% in the Communication sub‐domain.^33^ Similar data have been reported in independent cohorts^29^, ^30^ where 16p‐DEL carriers showed higher Social Interaction and Communication impairments compared with siblings.^29^ Socialization was significantly impaired when measured with the VABS as well but not consistently.^30^, ^34^, ^35^ Interestingly, Moreno‐De‐Luca et al. reported that the SRS score and the VABS Socialization score of 16p‐DEL were significantly correlated with those of their parents and siblings respectively, suggesting that parental ability to perceive, understand, communicate and their motivation to interact socially could be potential contributors to social impairments in their children.^34^ Similarly, Hudac et al.^36^ described a significant correlation between the VABS total score and perinatal events, with impaired adaptive abilities linked to perinatal complications (e.g., preterm labor, abnormal presentation, C‐section, respiratory distress, low APGAR score and/or low birthweight). A similar trend was observed for the ADOS social‐affective score (ADOS‐SA), measuring both Social Interaction and Communication.^36^ Overall, these findings show the presence of social impairments in a subset of 16p‐DEL carriers, involving both communication and social interaction, albeit with milder presentation in 16p‐DEL than in idiopathic autism (iASD) without CNVs.^33^, ^37^ Notably, the presence of social deficits in 16p‐DEL could be linked with perinatal complications and with the level of social responsiveness in their parents.

### 2.3 Social impairments in human 22q11.2 deletion carriers

Children with 22q11.2 deletion have been described as having poor social skills, including difficulties in initiation and attaining social relationships, shyness and poor relationships with peers^38^, ^39^ and these impairments exacerbate in late childhood.^40^ Rates of ASD in carriers of the 22q11.2 deletion (22q‐DEL) vary from 23%^33^ to 42%^9^ across studies, however higher percentages were reported in studies using the SCQ, varying from 26%^41^ to 91%.^42^ More reliable assessments for ASD,^43^ using both the ADI‐R and the ADOS, reported a lower 28% of ASD in 22q‐DEL.^44^ When the clinician evaluation was added to the interviews, as recommended by guidelines, a further reduction in ASD prevalence (16%–18%) was reported.^45^, ^46^

Taken together, these data indicate the presence of social and behavioral impairments in 22q‐DEL that may overlap with symptoms of ASD. Within the 22q‐DEL group, subjects with ASD (22q‐DEL + ASD) had more severe Socialization problems (measured with the VABS) than 22q‐DEL without ASD, and more social problems assessed with the CBCL, indicating poor quality of the interaction with peers.^9^ The ADOS‐SA differed between children with 22q‐DEL + ASD and 22‐DEL without ASD as well, indicating more severe impairments in 22q‐DEL + ASD than in 22q‐DEL without ASD in the sub‐domains of social interaction and communication.^45^ To better characterize these impairments and their link with 22q‐DEL (with or without ASD), carriers of this CNV were compared with patients with iASD. A less impaired social profile was observed in 22q‐DEL than in iASD, confirmed by both the ADI‐R and ADOS, and by the CBCL Withdrawal.^45^ Similarly, the ADOS‐SA was higher in the iASD group compared with the 22q‐DEL + ASD, who displayed relative strengths especially in nonverbal communication, including facial expression and gestures.^45^ However, the ADI‐R did not confirm these differences, with similar ADI‐R social interaction and communication scores in the iASD group and in the 22q‐DEL + ASD.^45^ Thus, such deficits might be classified as ASD symptoms, but they are milder and without the typical presentation. Taken together, these findings suggest that the 22q‐DEL + ASD could represent an intermediate social phenotype between iASD and 22q‐DEL (iASD >22q‐DEL + ASD > 22q‐DEL > general population).

To understand if some social sub‐domains are more impaired than others in 22q‐DEL, even without a clinical diagnosis of ASD, we looked for studies reporting the percentages of carriers scoring above the cut‐offs in the most relevant sub‐scales of the diagnostic tools (communication and social interaction). The sub‐domain of Communication measured with the ADI‐R was impaired in 32% of subjects with 22q‐DEL^45^, ^46^ and in 47% of the 22q‐DEL without ASD.^33^ Among 22q‐DEL with high total ADOS scores, impairments were mostly related to the Communication sub‐domain rather than social interaction.^47^

Impairments in the sub‐domain of Social Interaction were reported in 22q‐DEL children in percentages varying from 36%^46^ to 40%,^45^ as measured with the ADI‐R. Even after excluding subjects with a diagnosis of ASD from the sample, 46% of the remaining 22q‐DEL without ASD still presented impairments at the ADI‐R Social Interaction.^33^ Presence of Social Problems, assessed with the CBCL, were often reported in children with 22q‐DEL^38^, ^48^, ^49^, ^50^ ranging from 27% to 37%.^51^, ^52^, ^53^ Clinical elevations in the CBCL Withdrawal subscale was reported as a feature in 12%–14% of cases of 22q‐DEL children as well.^49^, ^53^ However, despite the deficits in this sub‐domain, it was suggested that 22q‐DEL children may still understand emotions and thoughts from others and may also be interested in social interactions.^44^ Notably, 22q‐DEL social impairments seem to intensify when growing older^50^, ^54^, ^55^, ^56^ and to correlate with deterioration of intellectual functioning, with greater deficits observed for lower IQ^57^ and for executive functions impairments.^58^ Thus, while the ability to adapt behavior to the context becomes increasingly important with age, deficits in the capability to correctly perceive,^40^ contextualize, process social information,^59^ and give adequate social responses, could determine the social problems observed in 22q‐DEL. These abilities are connected to the social‐cognitive domain of theory of mind, meaning the ability of perspective‐taking and abstracting feelings, thoughts, intentions of others from the context, during a social interaction.^60^ Deficits in theory of mind, assessed through the TASIT, have been consistently described in 22q‐DEL^61^, ^62^, ^63^ and have been linked to their risk of developing schizophrenia.^64^, ^65^ Considering that about 25% of 22q‐DEL carriers develop schizophrenia later in life,^66^ the link between social impairments and cognitive dysfunctions, and the progressive decline of social functioning after childhood, the presence of social deficits in 22‐DEL that are less severe than in iASD could represent a manifestation of vulnerability for psychotic disorders. Overall, the findings reported so far suggest more socio‐cognitive deficits in 22q‐DEL which tend to become exacerbated throughout development.

Although not limited to ASD, face identification and emotion recognition are other social‐cognition sub‐constructs extensively studied in 22q‐DEL, with contrasting results.^40^, ^67^, ^68^ Some studies have described impairments in recognition of faces in 22q‐DEL.^40^, ^69^ Moreover, 22q‐DEL were reported to have more problems properly identifying emotions compared with controls. Deficits in the recognition of angry, disgusted, fearful and neutral faces but not of happy, surprised and sad faces were described,^70^, ^71^ even though the results are not consistently replicated.^72^, ^73^, ^74^ Accuracy and reaction times in emotion recognition were measured in some studies, with opposing results.^54^, ^75^, ^76^, ^77^ Such discrepancies could be because of differences in the intensity of emotions presented during the tests, as 22q‐DEL carriers require more intense emotional cues to properly perform in these tasks.^78^ Moreover, they display less fixation events over face emotional stimuli^79^ and they seem to focus their gaze more on face regions less relevant for emotion recognition (such as mouth) rather than exploring more relevant regions such as eyes.^80^ Interestingly, 22q‐DEL show less impairment in face emotion recognition than iASD, but have poorer performance than people affected by schizophrenia, subjects with high psychotic risk and healthy family members.^69^, ^81^ Together, these results indicate that emotion recognition might add important information about social impairments in 22q‐DEL and on the longitudinal evolution of their psychopathology. We hypothesize that this could be true for 16p‐DEL as well, given that—to our knowledge—there are no studies on this. Indeed, a full social evaluation of CNVs carriers at risk of developing for neuropsychiatric disorders, which includes an emotion recognition assessment, could add relevant information to the specific profiles of social impairment for each CNV.

### 2.4 Comparisons of 16p11.2 and 22q11.2 social profiles

Comparing social profiles of different CNVs could aid our understanding of how different genetic variants contribute to specific deficits. Two studies have done this so far.^33^, ^82^ Cunningham et al.^82^ investigated the presence of emotional and behavioral abnormalities in 13 CNVs, including 16p‐DEL and 22q‐DEL, through the Developmental Behavior Checklist, an instrument specifically created to evaluate problems in intellectual disabilities. Although assessing social abnormalities in those two CNVs was not a specific purpose of the study (and the results were not discussed), the social phenotype of these two CNVs, compared with the others, seemed to present the mildest impairments, among all, in Communication, Social Interaction and self‐adsorption. Chawner et al.^33^ compared deletions and duplications at the 16p and 22q loci, with the purpose of characterizing the ASD profile of these CNVs and comparing ASD‐relevant impairments in the CNVs with iASD. 22q‐DEL seemed to be the least impaired in the Social Interaction and Communication sub‐domains of the ADI‐R. Generally, their profile was comparable with the one of 16p‐DEL, with the exception of socio‐emotional reciprocity and socially sharing of enjoyment, in which they displayed relative healthy functioning compared with 16p‐DEL. In any case, both the 16p‐DEL and 22q‐DEL were significantly less impaired in all the social constructs relevant for ASD, compared with the carriers of the duplications in the same loci, as well as compared with iASD. Interestingly, the Social Interaction impairments were less severe in 22q‐DEL when compared with other diagnostic groups displaying social deficits, such as patients with schizotypal personality disorder^83^ and comparable with those of people with idiopathic intellectual disabilities.^44^ The same comparisons were not available for 16p‐DEL.

The promising recent studies comparing CNVs reviewed above will hopefully be followed by more research using this approach. Indeed, as mentioned above, describing social profiles in CNVs carriers can lead to important insights on the interaction between genetics and behavior, and could address the determinants bridging the gap from genotypes to phenotypes. In fact, all the studies reported so far confirm that not all carriers of a CNV display social impairments. The cause of this variability of phenotypical effects within the same CNV is still unknown. It has been suggested that neurodevelopmental disorders could arise when presence of CNVs is conjoined with a second perturbing factor that adds its effect to the CNV‐related vulnerability.^84^, ^85^ Perturbing factors can be genetic “second‐hits,” such as simultaneous presence of another CNV or single nucleotide variations, or environmental factors such as maternal immune activation, perinatal events, and parental functioning. We previously mentioned that the presence of perinatal events and parental deficits in social functioning was correlated with higher social problems in 16p‐DEL.^34^, ^36^ Moreover, in 22q‐DEL, the presence of pro‐inflammatory cytokines has been linked with increased ASD‐related impairments, indicating a potential role of inflammation.^86^ In our opinion, mouse studies on CNV represent a promising model to investigate the genotype–phenotype gap, because they would enable the examination of in‐depth genetics and environmental factors as well as the biological mechanisms implicated in distinct social processes.

### 2.5 Preliminary considerations for studies in mice

Mice are the most commonly used animal models to study genetic alterations^87^, ^88^, ^89^ and several mouse lines with deletions of the syntenic 16p11.2 and 22q11.2 human regions have been generated (Table 2). The main goal of these mouse lines is to correlate altered behavioral phenotypes with brain circuits functioning and specific molecular/cellular pathways. However, studies on social profiling are still limited, especially in relationship to the assessments reported above in humans. In this section, we review the existing literature investigating social behavior in 16p11.2 and 22q11.2 deleted mouse lines, compare their profile, and highlight similarities and discrepancies with the literature describing human studies.

**TABLE 2:** Mouse models of 16p11.2 and 22q11.2 deletions reported in studies

16p11.2 deletion				
Model	Deleted region	Features	Genetic Background of the original mouse line	Genetic Background Tested in the experiment
*Mills* ^90^	*Slx1b‐Sept1* interval on mouse chromosome 7F4	*‐* Locomotor activity alterations (hyperactivity) ‐ Affected brain volume size and synaptic defects ‐ Sleep disorder ‐ Wider deletion than in humans (includes four genes outside the human BP4‐BP5 interval)	C57BL/6N:129Sv	‐ B6129S‐Del(7Slx1b‐Sept1)4Aam/J are generated from frozen sperm of df/+ mice mated to C57BL/6J mice for a minimum of two generations and used to fertilize B6129SF1/J oocytes.^91^ ‐ C57BL/6N129Sv^92^ ‐ C57BL/6 N^93^
*Dolmetsch* ^94^	*Coro1a‐Spn* interval on mouse chromosome 7F3	*‐* Hearing deficit (no particular hearing defect has been reported in humans with 16p11.2 CNVs)^95^	C57BL/6N	mixed background: 67% of C57BL/6N, 30% of 129P2/Ola, and 3% CD‐1^95^, ^96^, ^97^
*Herault* ^98^	*Sult1a1‐Spn* interval on mouse chromosome 7F3	‐ Homologous to human 16p11.2 BP4‐BP5 ‐ Created to overcome the limits of the two previous models *‐* Inbred C57BL/6N genetic background	‐ C57BL/6N ‐ F1 C57BL/6NxC3B	‐ C57BL/6N ‐ F1 C57BL/6NxC3B^98^
***22q11.2 deletion***				
**Model**	**Deleted region**	**Features**	**Genetic Background**	
*Df(16)A+/−* ^99^	Dgcr2‐Hira interval (1.3‐Mb) on mouse chromosome 16	‐ Syntenic to the 1.5‐Mb human 22q11.2 microdeletion ‐ Smaller dendritic spines ‐ Impaired sensorimotor gating ‐ Acquisition of a spatial working memory–dependent	C57BL/6J	C57BL/6J^100^
*Df1*/+^101^	Es2‐Ufd1l interval (∼1.2 Mb) on mouse chromosome 16	‐ Syntenic to 1.5 Mb region in human chromosome 22 ‐ Cardiovascular defects (are similar to those seen in patients) ‐ Hearing difficulty ‐ Impaired sensorimotor gating ‐ Deficits learning and remembering of complex cues^102^	‐ 129SvEvBrd (129S5) × C57BL/6	‐ C57BL/6^c−/c–^;129S5/SvEvBrd (mixed)^102^
*Lgdel/+* ^103^	Idd‐Hira interval (∼1.5 Mb) on mouse chromosome 16	‐ Syntenic to the 1.5‐Mb human 22q11.2 microdeletion ‐ Contains five more genes than in the Df1/+ mice of Df1/+ model—perinatal lethality ‐ Cardiovascular defects ‐ Conotruncal defects	C57BL/6 (substrain not reported)	LgDel129/Sv/C57BL/6/129S6/SvEvTac/FVB/N/SJL^104^
*Df(h22q11)/+* ^105^	*Dgcr2‐Hira* interval on mouse chromosome 16	‐ It encompasses orthologues of all functional genes on the critical human 22q11.2 locus with exception of clathrin heavy polypeptide‐like 1 (CLTCL) ‐ Increased NMDAr antagonist– induced locomotion only after puberty ‐ Decrease in sensorimotor gating ‐ Improved discrimination and reversal learning^106^	‐ C57BL/6N	‐ C57BL/6J^106^ ‐ C57BL/6NTac^107^
*Del(3.0 Mb)/+* ^108^	*Pi4ka ‐ Hira* interval (3.0 Mb) on mouse chromosome 16	‐ Reproduced the 3.0‐Mb 22q11.2 deletion that characterizes ~90% of patients differently from the other existing models with minor than 1.5‐Mb deletions ‐ Hypoactivity ‐ Impairment of rhythmic activity ‐ Behavioral flexibility	C57BL/6N	C57BL/6N^108^
Znf74‐Ctp^109^	*Znf74‐Ctp* interval (~150 kb) on mouse chromosome 16	‐ Increased PPI of the startle response.	Inbred 129/SvEv‐Tac (129S6) and mixed (129S6 × NIH Black Swiss)	Inbred 129/SvEv‐Tac (129S6) and mixed (129S6 × NIH Black Swiss)^109^

Like other mammals, mice show complex social behaviors as well as an elevated level of reciprocal social interactions.^110^, ^111^, ^112^, ^113^, ^114^, ^115^, ^116^, ^117^, ^118^ The numerous social tasks that have been developed and are currently available for mice, allow efficient assessment of communal nesting,^119^ sexual^120^ and parenting behaviors,^121^ territorial scent marking,^114^ reciprocal social exploration,^115^ aggressiveness,^112^ social hierarchy,^122^ social memory,^123^ social reward,^124^ social buffering,^125^ emotional contagion,^126^ emotion discrimination,^127^ cooperation,^128^ and many more. Despite this, 16p11.2 and 22q11.2 deletion models have only been assessed for sociability, social novelty, social memory, and ultrasonic vocalizations (USVs). Sociability and social novelty tasks are usually evaluated in a free social interaction setting or in a three‐chambered apparatus, characterized by three accessible zones (the chambers). In the “free social interaction” setting different mice are left free to interact with each other and the quantity and quality of their interactions is measured. In the three‐chamber tasks, the tested mouse is positioned in the central area of the apparatus and is allowed to explore the other two chambers containing two stimuli; either a mouse versus an objects or a familiar mouse versus an unfamiliar mouse. “Sociability” is defined as the willingness to spend time with another mouse, assessed during the mouse‐object discrimination phase or during free interaction.^110^, ^118^ This sociability construct has often been used to assess the lack of interest to spend time with others, often present in ASD. “Social novelty” describes the preference of a mouse to explore a novel mouse compared with a familiar one. The latter construct includes a “social memory” component that can be better studied by initial exposing the mouse to a conspecific and then re‐testing their interactions as a function of time.^129^ This ability is linked to social recognition and social learning,^130^ which form the basis of social relationships in both humans and animals.^131^ Analyses of USVs are often used as a measure of social communication in rodents. However, in contrast to rats, mice tend to vocalize primarily to solicit maternal care or during reproductive contexts, such as the exposure of a male to a female in estrus or to female urine.^132^, ^133^, ^134^, ^135^ USV recordings and analyses in CNVs have mostly been assessed in adult male mice during the courtship and mating interactions in male–female interactions.^95^

It should be noted that not all the studies used the same paradigms, and the same scoring method (manual vs. automated), even in the classical three‐chamber task.^110^ Moreover, each mouse line of the same CNV can have slightly different genetic changes (see Table 2) and importantly, different genetic background, which may influence e behavioral phenotypes through genetic deletion‐by‐genetic background interactions. Below, we group results according to the evaluated construct and note if different developmental stages or tasks were used.

### 2.6 Social impairments in mice 16p11.2 deletion carriers

The studies describing social behavior in 16p11.2 deleted mouse lines are still contradictory. Here, we summarize and assess available results to elucidate which factors can be important in their social profiling.

“Sociability” is the most investigated phenotype in 16p11.2 mice. No social impairment in adult *Dolmetsch* model as well as in adult *Mills* model was observed in the three‐chamber social interaction test.^91^, ^94^, ^96^ In addition, both models showed no deficits in reciprocal social interactions with no restriction and free possibility to interact at postnatal day (PND) 21–25.^91^, ^96^ Only Wang et al. reported sociability deficits in *Mills* 16p11.2 deletion mice.^92^ Notably, another investigation from Mitchell et al. reported that while in basal condition there were no differences in sociability between 16p11.2 and wild‐type mice, a genotype‐dependent impairment appeared following the stress of an injection.^93^ Overall, these studies highlight no overt sociability deficits in 16p11.2 mice in basal condition that could though appear in certain circumstances, emphasizing the importance of gene‐environmental interactions.

Social novelty scores have been reported normal in 16p11.2 mice of the *Mills* and *Dolmetsch* models.^91^, ^94^ In contrast, the 16p11.2 deletion *Herault* model showed a social preference deficit for the second stranger mouse in a three‐chambered social procedure.^98^ Similarly, the *Dolmetsch* line in a mixed C57BL/6N‐129P2/Ola‐CD1 background showed no discrimination for social novelty, even if increasing demonstrators‐related social cues (by using two different strains) a social novelty preference was evident.^97^ Thus, these data suggest that discrepant results could be related to the genetic background of the tested mice. For example, the *Herault* mouse line has a C57BL/6N × C3B background and the corresponding wild‐type mice show higher levels of basal social interaction.^98^ This suggests that for social deficits to manifest in 16p11.2 CNV carriers, a second “hit” (in this case genetic) may be needed. More consistent impairments were observed in 16p11.2 deletion mice in relationship to male–female interactions. In particular, in the *Dolmetsch* line, lower nose‐to‐nose sniffing duration, nose to nose sniffing bouts, nose to anogenital sniffing duration, nose to anogenital sniffing bouts and a lower number of ultrasonic vocalizations were recorded.^136^ Similarly, another study reported reduced anogenital sniffing and ultrasonic vocalizations, but no changes in nose‐to‐nose sniffing in the same model,^96^ during the first exposure to an unfamiliar estrous female as well as after the female was removed.^95^ In a subsequent experiment, the same research group showed the impact of genotype‐by‐housing factors. In particular, male–female interactions and USVs deficits in 16p11.2 *Dolmetsch* heterozygous mice were evident when cohoused with wild‐type animals but not when housed with only 16p11.2 heterozygous mice.^96^ However, Brunner et al., found that PND60 *Mills* male mice responded normally to female urine scent in an open field arena.^91^ This further suggest that basal social abilities are conserved in 16p11.2 deletion models, but that they are more vulnerable in showing social deficits following environmental stressful situations (e.g., physical encounter with a female with different genotype or injection related stress) with an influence of other genetic factors.

### 2.7 Social impairments in mice 22q11.2 deletion carriers

Sociability of Del(3.0 Mb)/+ were evaluated by the three‐chamber test showing no differences compared with WT mice.^108^ Similarly, another mouse model carrying the 22q11.2 microdeletion (i.e. Df(16)A+/− mice) showed no major sociability deficits during a first exposure to a conspecific.^100^ This was partially confirmed in Df(h22q11)/+) mutant mice which showed a mostly similar social repertoire compared with WT controls, although some alterations in social patterns of interactions were evident.^106^ In contrast, disruption of single genes within the 22q11.2 region (e.g., Sept5, a septin family member and Tbx1) in mice result in reduced levels of active social interactions.^137^, ^138^ Thus, further^92^ investigation are required to improve our understanding of basic social interaction abilities in 22q11.2 mouse models.

Clearer evidences are presented in relation to social novelty. Reduced reactions to social novelty have been consistently reported in Del(3.0 Mb)/ + mice,^108^ Df(16)A+/_ mice,^100^ as well as in LgDel mice.^104^ Notably, Saito et al. reported a normal pattern of habituation in a five trial free interaction test (in this task the subject mouse is exposed to the same mouse for four trials and to a novel mouse during the fifth trial) in Del(3.0 Mb)/ + mice when successively exposed to the same stimulus mouse, suggesting a more pronounced alteration in social novelty reaction, but conserved immediate social memory functions.^108^ However, an altered functioning of mouse hippocampal CA2, a region important for social memory, was found in Df(16)A+/− mice^100^, ^139^ strengthening an alteration of social memory processes in this genetic condition.

Notably, not all available 22q11.2 mouse lines have been screened for social behaviors. Moreover, for the majority of these mouse lines, a comprehensive investigation of their social profiling is still lacking, especially in terms of female–male interactions, ultrasonic vocalization and more human relevant socio‐cognitive abilities. In particular, as social dysfunctions in human subjects with 22q11.2 are distinct throughout development, and are linked to their cognitive functions, more socio‐cognitive abilities should be addressed throughout development in 22q11.2 mouse models. For example, this could include testing social processes such as emotion recognition, cooperation and emotional contagion or comparing their purely cognitive profile (e.g., executive functions, working memory and attention) with their social phenotype.

### 2.8 Social impairments in mice and humans: issues and future directions

As reported above, humans carrying 16p‐DEL and 22q‐DEL present socio‐cognitive deficits affecting both social interaction and communication. While 16p‐DEL display deficits in emotional reciprocity, 22q‐DEL appear to be less socially impaired at younger ages, but deteriorate when growing older, at a life stage when social interaction with peers requires more complex cognitive evaluations. To date, some deficits in social communication in 16p11.2 deletion mice have been reported, often in combination with other environmental and/or other genetic factors and while social behavior and cognition has not been fully examined in 22q11.2 deletion lines, these mice generally show differences in relation to social novelty interaction.

Comparing the available results from human and mice studies requires a certain level of approximation. The findings reported so far were obtained in two species with the purpose of answering different experimental questions. While mice are tested with the aim of quantifying and qualifying impairments in specific social sub‐constructs, such as sociability, social novelty and others, findings on social behavior in humans carrying 16p11.2 and 22q11.2 CNVs mostly rely on clinical instruments built in order to assess if carriers can be classified in specific diagnostic categories (i.e., ASD). Ideally, tests should be built to replicate the social domains described in the RDoC to facilitate comparability between studies and replication in animal models.

Another issue to keep in mind when comparing human and mice, is that they communicate socially with conspecifics using different modalities, which only partially overlap. Mice interaction between conspecifics, familiar and unfamiliar, male and female, is based primarily on olfaction, while auditory cues were mostly associated with mating and parenting.^140^ Whereas, in humans in addition to language and verbal communication, facial expression is an important channel of social communication,^141^ with specific brain areas and circuits dedicated to face recognition and to facial movement.^142^, ^143^ Interestingly, it has recently been showed that the visual cues may also be important in communication of affective states between mice,^127^, ^144^, ^145^ an ability comparable to the human emotion recognition.^135^ Facial and non‐facial communication, including face emotion recognition, are among the constructs that define social processes in the RDoC, and have been extensively studied in humans in the context of neuropsychiatric disorders.^146^ Considering that there is a growing body of literature showing that carriers of 22q‐DEL show impairments in face recognition and in emotion recognition, it would be relevant to study this domain in the animal counterpart in depth as well.

Although social characterization of 16p11.2 and 22q11.2 deletions in humans and animals is still in its early stages and needs further investigation, the results described so far are promising. Despite the limitations, research from both fields have provided evidence that social deficits are present in both humans and mice carrying these CNVs, and that they impact on both social interaction and social communication. In humans, not all carriers display significant levels of impairments, while in mice higher level of consistency have been observed, even when accounting for differences between laboratories and genetic backgrounds. Since mice are housed in controlled conditions, and studies have been mostly performed in inbred strains where genetic variability between subjects is low, it is possible that environment and genetics interact to determine the impairments which may explain why they are not evident in all carriers of these CNVs. The main aim of animal models is to provide a tool that will allow researchers not only to understand the role of specific genes in determining social impairments, but also enable them to intentionally interfere with the development of phenotypes, by modulating environmental factors in controlled conditions. We propose that future research in mice should address the study of factors that have already been linked with ASD‐like impairments in CNVs in humans, as reported in this review, namely parental behavior, perinatal events, and maternal immune activation. Identifying how a genotype leads to a specific phenotype through the contribution of environment may be crucial to address preventive interventions and to target therapeutics that can be translated into human studies.

## 3 CONCLUSIONS AND FUTURE DIRECTIONS

What contributes to the heterogeneity of behavioral phenotypes in 16p11.2 and 22q11.2 deletion carriers? Moreover, why do only some subdomains of social processes appear to be altered in these genetic disorders? We reviewed these two CNVs deletions in humans and animal models as we think that this might be a possible way to disentangle the complexity of social processes modulated by these genetic conditions.

Based on the available literature, social impairments in 16p11.2 and 22q11.2 deletions are clearly present. However, significant differences between and within the genetic conditions exist, especially in relation to social domain sub‐constructs. This is to be expected, as social processes are multilayers and complex and cannot be only addressed as a single “social interaction” measure. However, it is still unclear why not all subjects carrying a similar genetic condition (i.e. 16p11.2 and 22q11.2) would show similar behavioral alterations, especially in the context of mouse studies where the interference from other genetic and environmental factors is expected to be minimal. This conundrum requires dedicated research. Several genes are involved in 16p11.2 and 22q11.2 CNVs deletions, and data from single gene deletions are not always in line with data coming from the full CNVs deletions. One explanation could be that the increased interaction between multiple deleted genes contribute to the heterogeneity in behavioral phenotypes. Similarly, other genetic variants outside the CNVs region of interest might differently interact contributing to heterogeneous results between individuals with the same CNV. In this context, mouse studies combined with human genetic studies will be useful to disentangle potential epistatic effects (see e.g., References 147, 148).

Notably, from a clinical research point of view, some studies provided initial evidences that presence and severity of social behavior impairments are linked with environmental factors. Again, the advantage of being able to strictly control for environmental factors in mouse models could be important. Therefore, dedicated attention should be paid in relation to the genetic background strain, breeding and husbandry protocols, animal facilities conditions (the temperature and humidity of animal rooms, environmental enrichment, food, and others), social environment including pre and postnatal experiences, weaning age, and sex of the tested and stimulus subjects. In addition, wider arrays of social tests should be used in mice to better bridge the gap between clinical and preclinical research. This is particularly evident from the paucity of socio‐cognitive assessments employed so far in mouse models in contrast with human studies. Based on the literature, relevant tests could be emotion discrimination,^127^ emotional contagion,^126^ social personality traits,^149^ harm avoidance^150^ and altruism/selfishness paradigms.^151^

Emerging evidence suggests a meaningful crosstalk between the microbiota, the immune system and brain circuits in order to modulate social processes.^152^, ^153^, ^154^ This may be another environmental factor interacting with genetic CNVs conditions and ultimately contributing to their vulnerable and heterogeneous phenotypes. Indeed, microbiota composition can be very variable depending on diet and many other environmental factors: it was recently suggested that diversity in gut microbiome composition in people with ASD may be influenced by their diet, which is in turn impacted by their symptoms, such as restricted interests.^155^ Moreover, some associations between pediatric ASDs and various gastrointestinal symptoms such as constipation, diarrhea, food allergies, malabsorption or digestion problems are reported.^156^ Notably, germ free (GF) mice show sociability defects as they did not prefer to spend more time with a mouse compared with an empty chamber.^157^ Moreover, social deficits induced by maternal high‐fat diet can be rescued by supplementation with *Lactobacillus reuteri*, which has been associated with increased expression of oxytocin,^152^, ^158^ one of the main peptide hormones involved in social processes.^144^, ^159^ Intriguingly, Wu et al. recently reported that GF mice are characterized by reduced social activity toward novel stranger mice and show an increase in social activity after mono‐colonization with *Enteroccocus faecalis*, suggesting that specific gut microbiota populations can affect social behavior in mice.^160^ Overall, complexity and variability of diets and microbiome could be a candidate to explain the phenotypic variability reported in 16p11.2, 22q11.2 and other CNVs, which will deserve dedicated research.

Social connections are crucial factors influencing our life that implicate processes impacting the brain as well as the rest of the body.^152^ Considering this, individuals that are not able to establish proper contacts with others can have serious consequences, such as social isolation, non‐successful life or more pervasive neuropsychiatric conditions. Overall, we support the necessity to better comprehend socio‐cognitive processes, with a combined effort with more dedicated assessments in humans, paired with proper mechanistic and behavioral investigations in rodents and other animal models.

## Data Availability

Data sharing is not applicable to this article as no new data were created or analyzed in this study.
